# The Pathology of Appendicitis

**Published:** 1896-05-23

**Authors:** H. S. Wansbrough Jones


### THE PATHOLOGY OF APPENDICITIS.

By H. S. Wansbrough Jones, M.B. Edin., B.Sc., Lond.

It is only in the last few years that any satisfactory explanation of the pathology of appendicitis has been given. Even so lucid a writer as Talamano, whose book appeared in 1892, and whose views harmonised with those generally accepted at the time, offered as a solution of questions at issue theories which were not only incompatible with the limitations of anatomical possibility, but which did not acquire even the semblance of probability from any of the observations he recorded. His suggestion of "boulets fécals," manufactured by the churning which he supposed to take place in the cæcum and passed thence into the appendix, must be now held to be a quite untenable hypothesis, and there is no doubt that he also overlooked many of the anatomical considerations essential to a right understanding of the pathology of this condition.

It is not proposed to discuss the anatomy of the appendix in detail, but some few points are of such importance that they cannot be omitted. On transverse section it is found to be composed of the same structures as the cæcum, but with an excess of lymphoid tissue in the submucous coat, in some cases amounting to half the entire mass. It may be said to be composed of two tubes, a soft, distensible inner tube composed of mucosa and lymphoid tissue, and an outer inelastic tube composed of muscle and peritoneum.

It is supplied in most cases by a solitary terminal branch of the ileo-colic artery, but in some women receives a collateral supply by way of the appendiculo-ovarian ligament. The nerves are from the superior mesenteric plexus, and the veins lead to the superior mesenteric vein.

The position of the appendix in the abdominal cavity is subject to wide variations, and as it is so freely movable probably no importance should be attached to its relative position as such; it is only when it passes into some position where it may become strangulated or injured that this becomes of much moment. Its point of origin from the cæcum is in at least 90 per cent. of the cases on the postero-internal aspect of the cæcum just below the ileum, where it joins the large gut. This fact enables surgeons to find the appendix much more easily than any other.

The only other anatomical facts of importance are its relation to peritoneum and the existence of the peri-cæcal and retro-cæcal fossæ. The appendix is completely surrounded by peritoneum, and is attached to the ileum by a mesappendix. The fossæ named are formed by the junction of the ileum with the large intestine, and by the relation of the cæcum to the peritoneum lining the posterior abdominal wall, so that they are all lined by peritoneum. The peri-cæcal ones, the ileo-cæcal, and the ileo-colic are so small that it is almost impossible for a hernia of the appendix to take place into them. The retro-cæcal, on the other hand, is often found to contain the appendix, and this may lead to serious strangulation.

The contents of the appendix, as a rule, consist of mucus, with possibly a small amount of fæcal matter. If it be normally situated it can easily empty itself into the cæcum, though the facts that the orifice is extremely small, that its junction with the cæcum is oblique, and that experimentally it has been found difficult to make even water enter it from the cæcum, would seem to prove the improbability of the entrance of foreign bodies or even fæces, in anything but minute quantity, from the cæcum into the appendix. It is well known, however, that the appendix does often contain concretions which resemble rounded lumps of solid fæces, and it was to these that Talamano applied the term "boulets fécals," supposing that they were lumps of fæces that had been forced into the appendix. Now, apart from the difficulty of entrance that such solid bodies would encounter, and the fact that in the present day it is almost unknown to find foreign bodies, such as cherry stones, pins, &c.—which were supposed to be the common exciting causes of appendicitis—it has been definitely proved that the great majority of these fæcal concretions consist of inspissated mucus and salts, and are only coloured by fæcal matter. In some cases it is true they contain a large proportion of fat, but this is probably due to the breaking down of leucocytes. It is found also at times that they have as a core a small lump of fæces or even a foreign body. Berry, to whose excellent work I am indebted for many of the facts here given, found that a pultaceous mass was produced in the appendix as the result of ligature, and Hermann, by resecting a portion of gut, washing out the lumen, and then suturing the ends, found that a similar mass was produced as the result of intestinal secretion alone. There can be little doubt then that these fæcal concretions, or appendicular calculi, are formed in the appendix.

The mucus in the appendix naturally contains numerous micro-organisms, and it is in the action of some variety of these that the real exciting cause of appendicitis is to be found. Streptococci, the bacterium coli commune, the tubercle or typhoid baccillus and the fungus of actinomycosis have all been found in appendicular abscesses. Of these the two first are infinitely the commonest, either separately or in combination, and it is interesting to note in this connection that American observers have found that the temperature of patients suffering from appendicitis bears a direct relation to the number and activity of the streptococci present, and that where the inflammation is due entirely to the bacillus coli commune—the temperature is often normal, no matter how widespread the mischief. It is to the products of growth of the colon bacillus that the disgusting odour of appendicular abscesses is due.

It seems probable that future research will prove that tubercle is more commonly a cause of this disease than has been hitherto supposed. The well-known tendency of tubercle to affect lymphoid tissues supports this; there are also several cases on record where the tubercle bacillus was actually found in infected appendices. It has likewise been found in the appendix of a tubercular rabbit.

Typhoid appendicitis is extremely rare, and there is only one recorded instance of actino-mycotic appendicitis.

